# Robotic Right Hemicolectomy Provides Equivalent Oncologic Outcomes and Improved Perioperative Recovery Compared with Open Surgery

**DOI:** 10.3390/cancers18081310

**Published:** 2026-04-21

**Authors:** Hatice Altin, Thorsten Brechmann, Metin Mazgaldzhi, Anna-Marie Wilk, Benno Mann, Alexander Wilk

**Affiliations:** 1 Medizinische Fakultät, Ruhr-Universität Bochum, 44801 Bochum, Germany; hatice.altin@ruhr-uni-bochum.de (H.A.); thorsten.brechmann@rub.de (T.B.); m.mazgaldzhi@augusta-bochum.de (M.M.); mann@augusta-bochum.de (B.M.); 2 Department of Gastroenterology and Oncology, Knappschaft Kliniken Bottrop, 46242 Bottrop, Germany; 3 Clinic for General, Visceral and Robotic Surgery, Augutsa-Klinikum Bochum Mitte, 44791 Bochum, Germany; 4 English Divison, Faculty of Medicine, Wroclaw Medical University, 50-367 Wroclaw, Poland; annamarie.wilk@student.umw.edu.pl

**Keywords:** minimally invasive surgery, colorectal surgery, oncology, T4 carcinoma

## Abstract

**Background/objectives**: Open right hemicolectomy (ORH) remains the standard approach for locally advanced right-sided colon cancer. Minimally invasive techniques are increasingly applied in earlier disease stages; however, evidence regarding long-term oncologic outcomes in advanced tumors remains limited. This study aimed to compare the perioperative and oncologic outcomes of ORH versus robotic right hemicolectomy (RRH). **Methods**: In this single-center study, a prospectively maintained database of consecutive right hemicolectomy patients (2010–2020) was analyzed. The primary endpoint was overall survival (OS). Secondary endpoints included disease-free survival (DFS), perioperative outcomes, and histopathological quality. Additionally, a subgroup analysis was performed for T4 tumors. **Results**: A total of 198 patients was included, comprising 77 that underwent ORH and 121 that underwent RRH. RRH achieved oncologic outcomes comparable with ORH, with similar R0 resection rates (96% vs. 97.5%, *p* = 0.547) and lymph node yields (median of 18 nodes in both groups, *p* = 0.828). OS did not differ significantly (ORH, 45.3 months vs. RRH, 49.9 months, *p* = 0.130). DFS was longer in RRH (ORH, 42.2 months vs. RRH, 49.1 months, *p* = 0.029; HR = 0.575, 95% CI 0.349–0.947); however, this difference disappeared after adjustment for tumor stage. No survival advantage was observed in the T4 subgroup. **Conclusions**: RRH provides oncologic outcomes comparable to open surgery while offering perioperative advantages, even in locally advanced colon cancer. When performed in experienced high-volume centers, RRH represents a safe and oncologically sound alternative to ORH and may contribute to expanding MIC beyond early-stage disease.

## Simple Summary

Colorectal cancer is among the most common malignancies worldwide. Surgical resection remains a cornerstone of treatment. Traditionally, right-sided colon cancer has been managed with open surgery. Minimally invasive techniques are increasingly used in earlier disease stages. In recent years, robotic-assisted surgery has emerged as a minimally invasive alternative that may facilitate complex procedures. Its role in patients with more advanced tumors remains uncertain. In this study, we compared outcomes during and after surgery, resection quality, and long-term survival of robotic right hemicolectomy (removal of the right part of the large intestine) with a conventional open surgery group. Our findings demonstrate that robotic surgery, even in advanced tumor stage, maintains potential perioperative advantages over open surgery while achieving comparable oncologic results. When performed in high-volume centers, robotic surgery represents an oncologically sound alternative to open surgery and may facilitate the extension of minimally invasive surgery to patients with more advanced disease.

## 1. Introduction

Colorectal cancer (CRC) is one of the most common malignancies worldwide and represents a leading cause of cancer-related mortality. With more than 1.9 million new cases and over 900,000 deaths annually, CRC ranks among the three most frequent cancers globally and remains a major public health challenge [1,2,3]. In addition to multidisciplinary oncologic treatment strategies, surgical resection remains the cornerstone of curative therapy for localized and locally advanced disease. The substantial burden on public health systems underscores the importance of high-quality surgical treatment. Laparoscopy (LRH) has been the standard of care for right-sided colon cancer except for locally advanced tumors, in which surgical management still relies on open right hemicolectomy (ORH) [4,5]. Recent technological advances have challenged this standard approach by expanding the applicability of minimally invasive surgery (MIS) to complex oncologic and colorectal surgery, notably robotic surgery [6,7,8].

Robotic surgery offers several technical advantages over LRH, thereby addressing the key limitations of the laparoscopic approach, including three-dimensional visualization, hand-like instrument movements with increased wrist flexibility, and effective tremor reduction. By overcoming these limitations, robotic right hemicolectomy (RRH) facilitates the performance of technically demanding procedures and may have the potential to expand the indications for MIS to more complex procedures [9,10,11,12,13]. Despite hypothetical advantages, the current evidence in right-sided hemicolectomy regarding clinical and oncologic benefit remains limited. Selected studies investigating right-sided hemicolectomy have reported potential associations between RRH and improved clinical outcomes compared with open or laparoscopic approaches without allowing conclusions regarding oncologic superiority [9,10,14,15]. Consequently, current international guidelines recommend MIS as the preferred approach for colon cancer when performed by experienced surgeons. Regarding perioperative and oncologic safety, RRH and LRH are considered equivalent [16,17,18].

Nevertheless, ORH remains a widely utilized approach, particularly in patients with locally advanced tumors, allowing for effective management in cases of organ-transcending growth or multivisceral involvement [5]. Laparoscopy remains inherently limited in these situations [13,19]. Preliminary evidence indicates that technical feasibility may be expanded in selected clinical scenarios through RRH [20].

The role of the robotic approach in right-sided hemicolectomy, particularly in more advanced tumor stages, has not yet been conclusively established. Accordingly, further robust data is required to draw reliable conclusions regarding perioperative and oncological outcomes. Therefore, the aim of the present study was to compare robotic with open right hemicolectomy based on a prospectively maintained institutional database, including a subgroup analysis of patients with T4 carcinomas, with a particular focus on long-term oncologic outcomes. Against this background, we hypothesized that robotic right hemicolectomy achieves long-term oncologic results comparable to, or exceeding, those of open right hemicolectomy.

## 2. Materials and Methods

### 2.1. Study Population

**Figure 1:**
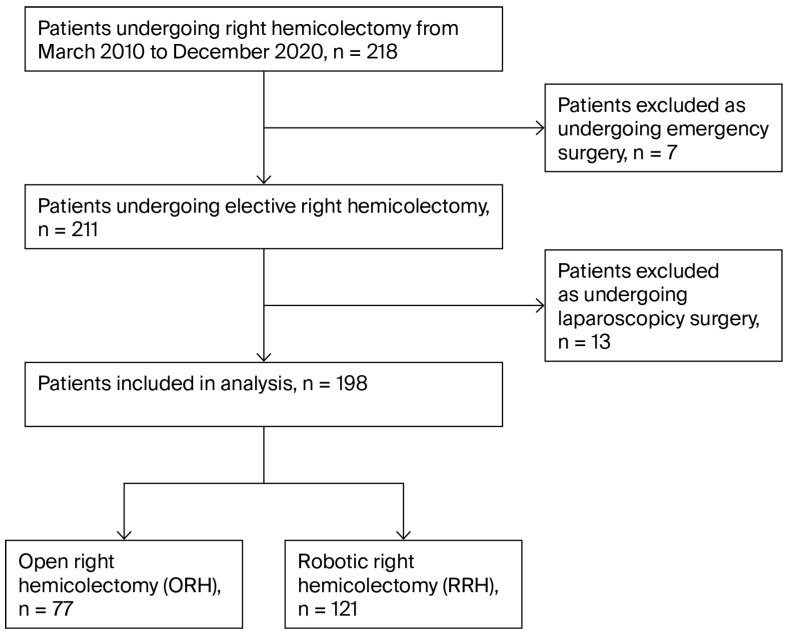
Flowchart of patient inclusion.

The institutional, prospectively collected, colorectal surgery database was retrospectively analyzed in a single-center observational cohort study. Consecutive adult patients who underwent either open or robotic right-sided hemicolectomy at Augusta Academic Hospital Bochum, Germany, between March 2010 and December 2020, were included. A conventional or robotic surgical approach was employed between 2010 and 2015, whereas from 2015 onward, all procedures were primarily initiated as robotic surgeries. In selected cases, deviations from this standard approach may have occurred due to the extent of tumor involvement. Additional accompanying abdominal surgery was allowed. The type and extent of surgical resection was assigned by a multidisciplinary tumor board considering the location, entity, and overall clinical status of the patient. All patients who underwent a laparoscopic approach were excluded due to the small sample size (n = 13), as well as those that underwent emergency surgery (Figure 1).

### 2.2. Data Collection

Surgical data were collected prospectively and maintained from patient records in the clinic’s database. Demographic characteristics included age, sex, body mass index (BMI), ASA classification (American Society of Anesthesiologists), prior abdominal surgeries, preoperative laboratory values, and peridural catheter placement. Characteristics of the surgical procedure included the duration of the operation, blood loss, blood transfusions, intraoperative complications according to the ClassIntra classification, conversion rate, need for additional resection, restoration of bowel continuity via an anastomosis, stoma creation, and devices used during surgery.

Postoperative parameters included the length of stay in the intensive care unit, as well as the total length of stay, complication rates according to the Clavien–Dindo classification (I–V), relaparotomy rate, anastomotic leakage, and 30-day mortality. Specifics regarding the administration of perioperative chemotherapy were retrospectively obtained from patient records and the institutional oncologic database.

Histopathological analysis encompassed the entity, R-status, UICC classification (Union for International Cancer Control), tumor size, ECOG classification (Eastern Cooperative Oncology Group), and the number of harvested and affected lymph nodes.

### 2.3. Primary and Secondary Objectives

The primary objective was five-year overall survival (OS) for ORH and RRH. Secondary endpoints included disease-free survival (DFS), technical quality parameters, perioperative parameters, and histopathological quality.

### 2.4. Statistical Analysis

The study population was divided into two groups based on the surgical procedure performed. Statistics were realized with SPSS version 31.0.0.0 (117) (IBM SPSS Statistics License: IBM SPSS Statistics). Normality was assessed using the Kolmogorov–Smirnov test and was not met (*p* < 0.001). Non-parametric methods were therefore used for inferential statistics. Effect sizes were calculated online [21]. Cohen’s d and Phi were used to quantify the effect size of the difference between two metrical or nominal variables, respectively [22]. For the analysis of long-term survival, the Kaplan–Meier method was applied, and to quantify the influence of specific variables on survival time, Cox proportional hazard regression and adjusted Cox regression were calculated. An intention-to-treat analysis was performed. Patients lost to follow-up were conservatively classified as having experienced disease recurrence: when no additional information on vital status was available during follow-up, overall survival was censored at the time of disease recurrence. The median duration of follow-up was 60 months. A binomial logistic regression was performed to determine the effect of certain variables to predict the likelihood of overall survival and disease-free survival. A separate subgroup analysis of patients with T4 carcinomas was conducted.

### 2.5. Definitions

The operation time was defined as the period between the skin incision and the completion of skin suture. Blood loss (BL) was determined as the measured volume within the suction device at the end of the operation. A conversion during robotic surgery was defined as any laparotomy performed for reasons other than specimen retrieval. Combined multi-organ resections were classified as minor and major. Minor resections included biopsy excisions, atypical liver resections, or intestinal suture. Major resections involved partial or complete removal of additional organs. Intraoperative complications were classified according to the ClassIntra classification, with major complications being defined as grade 3 or higher [23].

Postoperative complications were classified according to the standardized Clavien–Dindo classification, with major complications being those classified as grade 3 or higher [24]. Anastomotic leakage was categorized according to the ISREC classification into three grades (A–C) [25]. Histological resection status was defined according to the TNM classification [26]. Locally advanced colon cancer was defined as T4 (T4a/T4b) disease according to the AJCC TNM staging, eighth edition [27].

### 2.6. Operation Procedures

Robotic and open right hemicolectomy with complete mesocolic excision (CME) were performed following the oncological technique described by Hohenberger et al. [28] and Strey et al. [29]. Operative procedures were performed by five experienced colorectal surgeons. All of them performed open right hemicolectomy (ORH), while three performed robotic right hemicolectomy (RRH). All surgeons were board-certified specialists in visceral surgery. The surgeons performing RRH had passed the institutional credentialing process for robotic colorectal procedures.

The robotic procedures were conducted using the da Vinci X system. Patients were positioned supine with a mild Trendelenburg tilt and a slight left tilt. Capnoperitoneum was established via Palmer’s point. Four 8 mm robotic ports were inserted in an oblique arrangement between the left costal margin and the right inguinal ligament, with at least 8 cm spacing between them. Additionally, one assistant port (2 mm) was placed in the left mid-abdomen. Following identification and preparation of the mesenteric fold containing the ileocolic vessels, central ligation was performed using Hem-o-lok clips (Polymer ligating clips (Weck^®^ Hem-o-lok^®^, Teleflex Incorporated, Wayne, PA, USA)). Lymphadenectomy was performed along the superior mesenteric vessels in accordance with CME principles. The perfusion of the remaining intestine was assessed using indocyanine green and immunofluorescence. The transverse colon and terminal ileum were transected using a robotic stapler. Subsequently, an intracorporeal hand-sewn ileocolic anastomosis was performed as a side-to-side ileotransversostomy. After completion of the anastomosis, the resected specimen was extracted through a suprapubic Pfannenstiel-like incision.

For open right hemicolectomy (ORH), a midline laparotomy was performed. The right colon and terminal ileum were mobilized via lateral-to-medial dissection, maintaining the integrity of the mesocolic fascia. Mobilization was initiated along the white line of Toldt and continued in the embryological plane towards the hepatic flexure and duodenum, with separation from retroperitoneal structures. This lateral-to-medial approach represents the standard technique at our institution, allowing early mobilization of the right colon and facilitating dissection in cases with intraabdominal adhesions. In selected cases, particularly in locally advanced tumors, a medial-to-lateral approach with early central vascular control was applied at the surgeon’s discretion. Central vascular ligation of the ileocolic vessels and en bloc lymphadenectomy along the superior mesenteric vessels were performed. After exposure of the mesenteric root, the ileocolic vessels were identified and ligated at their origin. Lymphadenectomy was extended along the superior mesenteric vein and artery in accordance with complete mesocolic excision (CME) principles. The terminal ileum and transverse colon were transected, after which the specimen was retrieved. Reconstruction was performed via hand-sewn side-to-side ileotransversostomy. Routine placement of a drain was not performed in both procedures.

### 2.7. Ethical Approval

The study protocol was approved by the ethics committee of Westfalen–Lippe (registry number 2025-454-f-S) in accordance with the ethical guidelines of the Declaration of Helsinki and its subsequent revisions. Informed consent was obtained from all patients prior to undergoing surgery for subsequent processing and storage of data.

## 3. Results

### 3.1. Demographic Characteristics

A total of 198 patients was included, comprising 77 patients in the open surgery group (ORH) and 121 in the robotic surgery group (RRH). As displayed in Table 1, both groups were comparable with respect to age, gender, body mass index (BMI), American Society of Anesthesiologists (ASA) score, ECOG performance status (Eastern Cooperative Oncology Group) (Table S1) and UICC tumor stage, as well as the prevalence of vascular comorbidities, prior abdominal surgery, and preoperative laboratory parameters (Table S2). Differences were observed regarding the proportion of T4 tumors (ORH: n = 28 (36.4%) vs. RRH: n = 26 (21.8%); χ^2^ (1, N = 54) = 4.934; *p* = 0.026; Phi = 0.159; Table 2).

### 3.2. Characteristics of the Surgical Procedure

No significant differences were observed between ORH and RRH regarding the placement of peridural catheters (PDCs), intraoperative blood loss, the need for intraoperative blood transfusions, or the incidence of intraoperative complications (Table 1). RRH showed a significantly longer operative duration (ORH: 199 min [IQR 99] vs. RRH: 215 min [IQR 93]; U = 2994; Z = −2.997; *p* = 0.003; Cohen’s d = 0.451).

Notable differences were observed in the proportion of additional minor and major resections. The open surgery group showed a significantly higher number of patients who underwent additional minor resections (ORH: n = 16 (21.3%) vs. RRH: n = 10 (8.6%); χ^2^ (1, N = 26) = 6.227; *p* = 0.013; Phi = 0.181) and major resections (ORH: n = 18 (24%) vs. RRH: n = 11 (9.5%); χ^2^ (1, N = 29) = 7.454); *p* = 0.006; Phi = 0.198) (Table 1).

### 3.3. Postoperative Characteristics

Table 3 shows that the overall length of hospital stay (LOS) was significantly shorter in RRH than in ORH (ORH: median of 17.0 days [IQR 9.0] vs. RRH: median of 13.0 days [IQR 8.0]; U = 2961.5; Z = −4.182; *p* < 0.001; Cohen’s d = 0.626). The incidence of major complications was higher in ORH (ORH: n = 17 (22.1%) vs. RRH: n = 14 (11.7%); χ^2^ (1, N = 31) = 3.834; *p* = 0.050; Phi = 0.14) (Table 3). However, no significant differences were observed regarding surgical, non-surgical, or minor complication rates. Furthermore, both cohorts demonstrated similar outcomes regarding blood transfusions in total, 30-day mortality, and re-operation rates (Table 3 and Table S3).

### 3.4. Histopathological Results

In the robotic group, 113 patients had a histologically confirmed carcinoma compared with 76 patients in the open surgery group (ORH: n = 76 (98.7%) vs. RRH: n = 113 (93.4%); χ^2^ (1, N = 189) = 5.305; *p* = 0.021; Cohen’s d = 0.300, Table 2). A complete resection (R0) was achieved in 95.5% (n = 189) of cases overall, with 96% in ORH (n = 72) and 97.5% in RRH (n = 117) (H (1) = 0.362, *p* = 0.547). The median tumor size was 55 mm [IQR 28.5] in ORH compared to 45 mm [IQR 29.0] in RRH (U = 3218.5; Z = −1.633; *p* = 0.102). The number of harvested lymph nodes was identical between the open and robotic procedures (ORH: n = 18 [IQR 9] vv. RRH: n = 18 [IQR 9]; U = 4394; Z = −0.217; *p* = 0.828) (Table 2).

### 3.5. Survival Analysis

**Figure 2:**
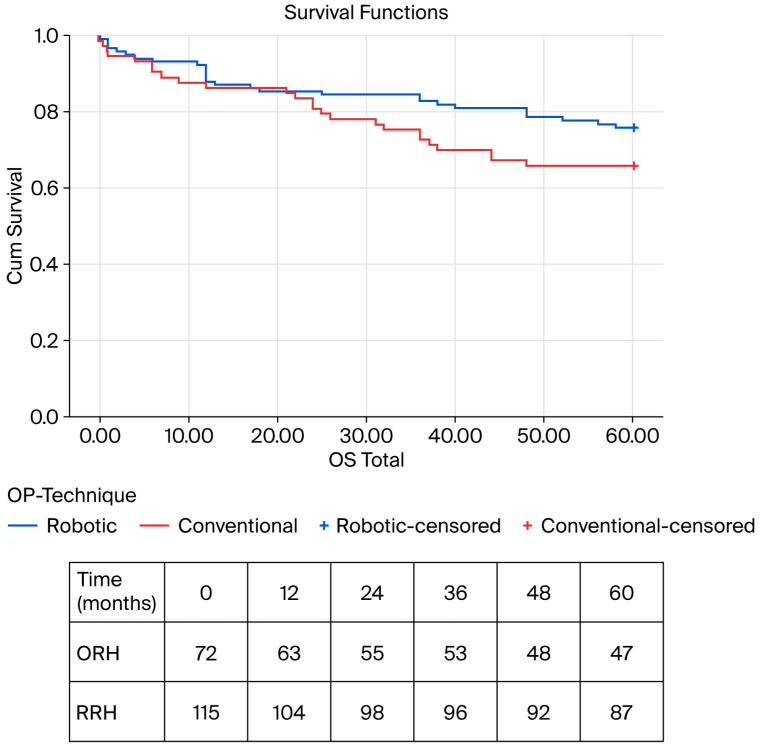
Kaplan–Meier survival analysis for OS—overall cohort; ORH: 45.3 months [95% CI 40.4–50.1] vs. RRH: 49.9 months [95% CI 46.4–53.5]; *p* = 0.13. OS—overall survival, ORH—open right hemicolectomy, and RRH—robotic right hemicolectomy.

**Figure 3:**
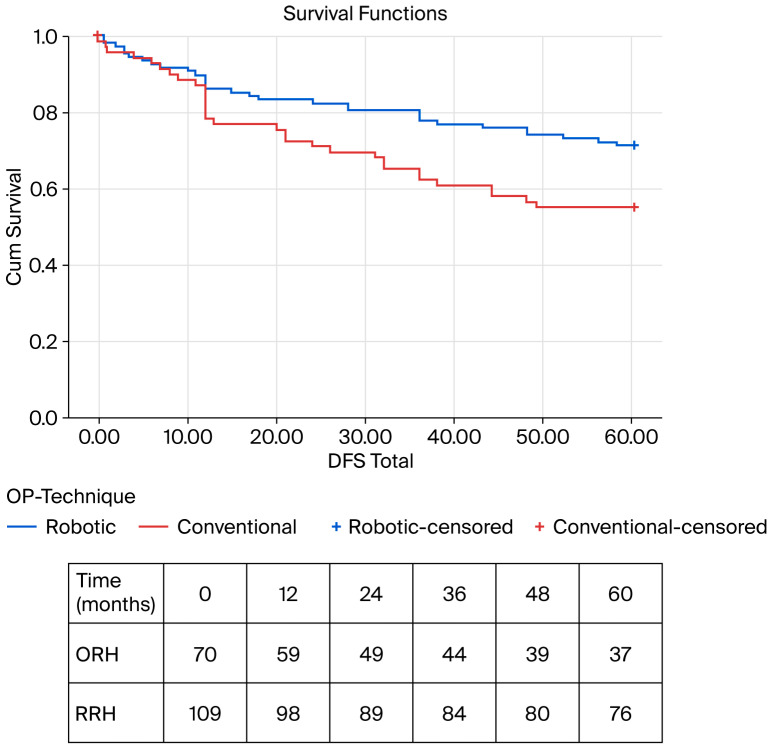
Kaplan–Meier survival analysis for DFS—overall cohort; ORH: 42.2 months [95% CI 37.1–47.4] vs. RRH: 49.1 months [95% CI 45.4–52.8]; *p* = 0.029; Cohen’s d = 0.328. DFS—disease-free survival, ORH—open right hemicolectomy, RRH—robotic right hemicolectomy.

Overall survival (OS) showed nominal differences between the groups (Kaplan–Meier: ORH, 45.3 months [95–CI 40.4–50.1] vs. RRH, 49.9 months [95–CI 46.4–53.5]; *p* = 0.130; Table 2 and Figure 2). In contrast, disease-free survival (DFS) was significantly prolonged in the RRH group (Kaplan–Meier: ORH, 42.5 months [95–CI 37.1–47.4] vs. RRH, 49.1 months [95–CI 45.4–52.8]; *p* = 0.029; Cohen’s d = 0.328; Table 2 and Figure 3).

Cox regression analysis demonstrated a 42.5% lower risk of recurrence in the RRH cohort (HR = 0.575 [95–CI 0.349–0.947]; χ^2^ (1) = 4.732; *p* = 0.030). Multivariable Cox proportional hazard regression analysis identified R-status as the only variable independently associated with OS, with R1 resection being associated with significantly worse survival (HR = 0.19 [95–CI 0.060–0.610]; χ^2^ (1) = 7.87; *p* = 0.005). No significant associations were observed for T4 tumor stage, surgical approach, or additional surgical procedures (Table S4). After stratification by surgical technique, R-status remained an independent predictor of OS (HR = HR = 4.80 [95–CI 1.05–22.02]; χ^2^ (1) = 4.080; *p* = 0.043; Table S5). With respect to DFS, before stratification, multivariable Cox regression identified T4 tumor stage (HR = 0.38 [95–CI 0.21–0.69]; χ^2^ (1) = 10.251; *p* = 0.001), minor additional surgical procedures (HR = 0.48 [95–CI 0.23–0.99]; χ^2^ (1) = 3.925; *p* = 0.048), and major additional procedures (HR = 0.33 [95–CI 0.17–0.65]; χ^2^ (1) = 10.195; *p* = 0.001) as independent predictors (Table S6). After stratification by surgical technique, T4 tumor stage (HR = 3.08 [95–CI 1.46–6.48]; χ^2^ (1) = 8.786; *p* = 0.003) and major additional surgical procedures (HR = 3.24 [95–CI 1.47–7.14]; χ^2^ (1) = 8.526; *p* = 0.004) remained independently associated with DFS, whereas minor additional procedures did not retain statistical significance (Table S7).

### 3.6. Binary Regression Analysis

A binomial logistic regression was performed to determine the effect of BMI, ASA, ECOG, additional procedures, major postoperative complication, chemotherapy, T4 carcinoma, affected lymph nodes, R-status, and tumor size in predicting the likelihood of OS and DFS. The binomial logistic regression model was statistically significant for OS, χ^2^ (11) = 30.49, *p* = 0.016, and DFS χ^2^ (11) = 46.47, *p* < 0.001, resulting in an acceptable explained variance by Nagelkerke’s *R*^2^ = 0.318 and in a medium amount of explained variance, as shown by Nagelkerke’s *R*^2^ = 0.44, respectively [30]. For OS, the overall percentage of accuracy in classification was 76%, with a sensitivity of 31.4% and a specificity of 94.2%. The overall percentage of accuracy in classification for DFS was 78.6%, with a sensitivity of 44.4% and a specificity of 92.2%. Of the eleven variables entered into the regression model, no variables significantly impacted overall survival, while two contributed significantly to predicting disease-free survival: major additional procedure (*p* = 0.005) and T4 carcinoma (*p* = 0.004). Patients with T4 tumors and major additional procedures showed odds ratios (ORs) of 7.03 (95–CI 1.85–26.71) and 12.69 (95–CI 2.17–74.30), respectively (Tables S8 and S9).

### 3.7. Subgroup Analysis

**Figure 4:**
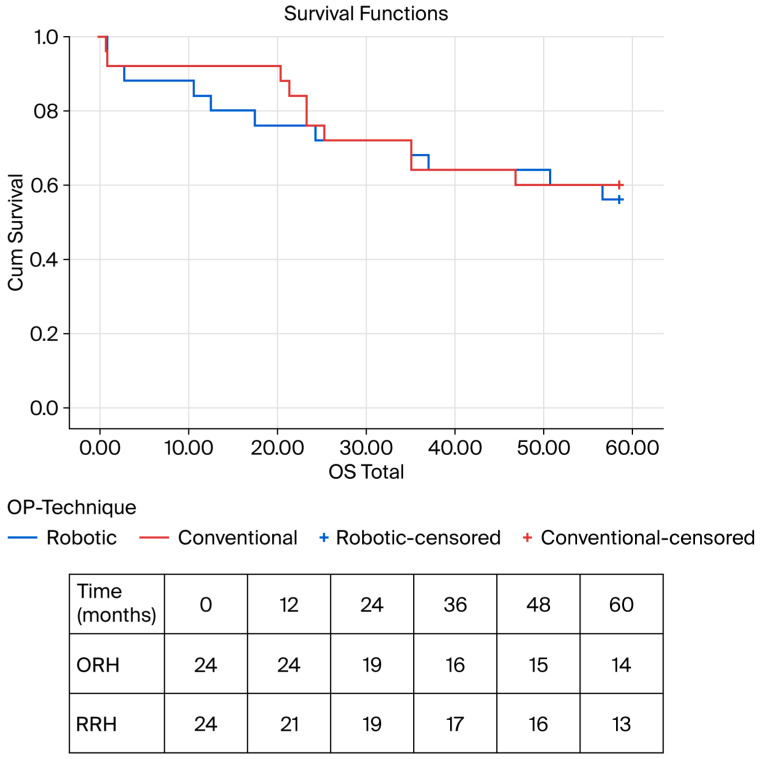
Kaplan–Meier survival analysis for OS—T4 subgroup; ORH: 60 months [IQR 35.0] vs. RRH: 60 months [IQR 38.5]; *p* = 0.77; chi-square = 0.083; *p* = 0.773. OS—overall survival, ORH—open right hemicolectomy, RRH—robotic right hemicolectomy.

The subgroup analysis of patients with T4 tumors revealed a longer overall hospital stay (LOS) in the open surgery cohort (ORH: 20 days [IQR 12] vs. RRH: 14 days [IQR 22]; U = 509; Z = 0.033; *p* = 0.012; Cohen’s d = 0.727). No differences were observed regarding demographic, perioperative, and histopathological parameters (Table 4). No significant differences were observed regarding minor (ORH: n = 9 (32.1%) vs. RRH: n = 4 (16%); *p* = 0.173; χ^2^ (1, N = 13) = 1.859) and major additional procedures (ORH: n = 10 (35.7%) vs. RRH: n = 6 (24%); *p* = 0.354; χ^2^ (1, N = 16) = 0.860). The subgroup analysis and an additional Mantel–Cox log-rank test showed no differences in long-term survival regarding OS (ORH: 60 months [IQR 35.0] vs. RRH: 60 months [IQR 38.5]; *p* = 0.770; log-rank χ^2^ (1) = 0.083; *p* = 0.773; Figure 4) and DFS (ORH: 26 months [IQR 48.0] vs. RRH: 40.5 months [52.2]; *p* = 0.797; log-rank χ^2^ (1) = 0.031; *p* = 0.861; Figure S1) (Table 4 and Table S10).

## 4. Discussion

This single-center observational cohort study showed that robotic right hemicolectomy (RRH) achieved similar oncologic outcomes compared with open surgery, while offering perioperative advantages, particularly in patients with locally advanced disease.

In our primary analysis, OS was comparable between the RRH and the ORH cohorts, whereas DFS appeared to be significantly longer in the robotic group. Importantly, histopathological and oncological quality parameters, including R0 resection rates, tumor size, and the number of harvested lymph nodes, as well as demographic characteristics, especially ASA and ECOG scores, UICC stage, or age, were largely comparable between both cohorts, suggesting that the observed DFS difference was not driven by differences in surgical radicality, specimen quality, or baseline patient characteristics, and no relevant differences in oncologic surgical quality were present between the two approaches.

However, the proportion of locally advanced T4 tumors was significantly higher in the ORH cohort, representing a major imbalance in disease severity and a relevant confounding factor for the observed difference in DFS. After adjustment in the T4 subgroup analysis, these differences in DFS were no longer evident. In this context, the robotic approach demonstrated non-inferiority with respect to oncologic outcomes. The initially observed DFS benefit in the overall cohort likely reflects the inclusion of patients with less advanced disease in the RRH group rather than the true effect of the surgical approach, particularly considering the unequal distribution of T4 tumors between the groups. Multivariable and binary regression analyses strongly support this interpretation, showing that tumor-related and procedural factors, particularly T4 tumor stage and the need for additional major resections, were the strongest independent predictors of DFS.

On the other hand, with respect to several parameters, RRH presented a more favorable outcome. For instance, within the T4 subgroup, patients undergoing RRH had a shorter LOS and a nominally lower rate of major postoperative complications compared with those undergoing ORH, indicating a clinically relevant perioperative benefit of the robotic approach in locally advanced disease.

Beyond perioperative outcomes, oncologic survival analyses provide further insight into the determinants of long-term prognosis. In our multivariable analysis, R-status was identified as the only independent predictor of overall survival. This finding underscores that tumor-related factors remain the primary determinants of long-term oncologic outcomes. In this context, the surgical approach appears to play a subordinate role regarding survival and predominantly influences perioperative outcomes.

The results of this study align with the growing body of evidence supporting the oncologic safety and efficacy of robotic-assisted colorectal surgery, even in more advanced tumor stages [5]. While the short-term benefits of the minimally invasive approach regarding perioperative recovery are well-established, demonstrating oncologic equivalence has been a critical subject of debate. Regarding short-term perioperative outcomes, a substantial body of evidence has consistently demonstrated that robotic-assisted colorectal surgery is associated with favorable or at least non-inferior perioperative results compared with open and laparoscopic approaches, as partly reflected in our findings. Across multiple studies and meta-analyses, these benefits include reduced intraoperative blood loss, higher lymph node yield, lower conversion rates, reduced urinary complications, faster recovery of bowel function, decreased postoperative morbidity, and shorter LOS despite operative times that are often longer [10,14,15,31]. Regarding OS and DFS, our data clearly suggest an oncologic non-inferiority of the robotic procedure, consistent with long-term results of the randomized controlled trial by Park et al., which reported no significant differences in five-year survival rates between minimally invasive and open resections [32]. Similarly, Spinoglio et al., in a consecutive series of RRH with CME, reported excellent five-year survival rates, underscoring the oncologic safety of the technique [33]. Our observation that there were no significant differences in lymph node yield or R0 resection between the open and robotic approaches is consistent with findings from large meta-analyses by Xu et al. and Trastulli et al. [10,13]. The number of harvested lymph nodes in both cohorts exceeded the guideline-recommended threshold of a minimum of 12 lymph nodes, reflecting the adequate quality of lymphadenectomy in accordance with oncologic principles [16,17,18].

With robotic surgery, financial evaluations must be taken into consideration. A formal cost analysis was not available in the present study. However, previous studies and meta-analyses consistently report costs for robotic colorectal surgery compared with alternative procedures. This is mainly driven by increased equipment costs, longer operating times, and technical support. Nevertheless, cost-effectiveness is not solely determined by the surgical platform itself but is influenced by perioperative outcomes, and financial compensation shows variability across healthcare systems. For this reason, complete treatment pathways need to be taken into consideration, possibly favoring robotic surgery. Since this is an ongoing debate, further research is to be conducted [10,34,35,36,37].

Despite the study’s profound methodology and the clinically relevant findings observed, several important limitations of the study must be acknowledged and should be considered when interpreting the results. First, the retrospective analysis of a prospectively maintained single-center database inherently limits the level of evidence and restricts the generalizability of the findings. Although data collection was standardized, retrospective analyses remain susceptible to incomplete documentation, information bias, and residual confounding that cannot be fully addressed by statistical adjustment. In addition, the small sample size may further limit statistical power. Second, the non-randomized study design introduces a relevant risk of selection bias. This is particularly important given the temporal shift in surgical strategy at our institution, with an increasing predominance of robotic procedures after 2015. The choice of surgical approach may also have been, at least in part, influenced by tumor characteristics and disease severity. Over the long inclusion period, improvements in perioperative management, anesthesia, postoperative care, enhanced recovery after surgery (ERAS) implementation, and oncologic treatment strategies may have occurred and could have influenced outcomes independently of the surgical approach. The coincidence of both factors, namely the temporal shift in surgical strategy and improvements in perioperative care, may have introduced a temporal bias, potentially confounding perioperative. Although multivariable analyses and subgroup analyses were performed to mitigate these effects, unmeasured confounders cannot be excluded. Another important limitation relates to follow-up completeness. A proportion of patients was lost to follow-up, which may have introduced bias in the assessment of long-term oncologic outcomes, particularly DFS. Patients lost to follow-up were conservatively classified, reducing the risk of overestimating DFS. While this intention-to-treat-like approach reduces the risk of falsely favorable outcomes, it may conversely lead to an underestimation of true DFS and potentially attenuate differences between groups. Third, laparoscopic right hemicolectomy was excluded from the analysis due to a small number of cases, which precludes a three-arm comparison between open, laparoscopic, and robotic techniques. Consequently, the results only allow conclusions regarding equivalence or non-inferiority between open and robotic surgery and do not permit direct benchmarking of robotic surgery against the standard. Finally, this study was conducted in a high-volume academic center with extensive experience in robotic surgery. At the Augusta Academic Hospital Bochum, robotic surgical systems have been in clinical use since 2010, reflecting early institutional experience. At our institution, robotic systems are applied not only in colorectal surgery but also across multiple other surgical disciplines, including hepatobiliary surgery [38]. Surgical expertise, institutional volume, and standardized CME techniques may have contributed substantially to the favorable outcomes observed in the robotic cohort. The potential influence of the institutional learning curve should be interpreted in this context, as robotic surgery has been continuously developed since its introduction at our institution, without exclusion of early cases. Thus, the reported outcomes reflect real-world institutional experience from the initial implementation phase onward. While this may limit direct comparability to centers with different learning trajectories, it also indicates that the observed results were achieved during an ongoing learning process.

Furthermore, the economic impact and cost-effectiveness of robotic surgery remain insufficiently defined, which may limit its broader implementation in routine clinical practice.

Taken together, these limitations need to be considered when interpreting the results; however, they do not diminish the relevance of the present findings. Beyond the existing prospective randomized evidence, this investigation adds substantial clinical value by providing long-term outcome data from a large patient cohort and reflecting routine clinical practice in a high-volume center, thereby enhancing external validity and real-world applicability. The present findings demonstrate that RRH can be performed with consistent oncologic quality and favorable perioperative outcomes, even in a cohort with a substantial proportion of locally advanced tumors. In particular, the dedicated analysis of T4 carcinomas adds important evidence to an area where data remain limited and treatment strategies are still evolving. By highlighting the strengths and boundaries of robotic surgery in advanced disease, this work contributes meaningful clinical insight to the ongoing discussion on patient selection and surgical strategy in right-sided colon cancer. In this context, our findings support a differentiated, experience-dependent approach to surgical decision-making rather than a stage-based exclusion of minimally invasive techniques.

## 5. Conclusions

In summary, this study demonstrates that robotic right hemicolectomy provides equivalent oncologic outcomes and partly improved perioperative recovery compared with open surgery. These findings are consistent with the growing body of evidence supporting robotic colorectal surgery, although data specifically addressing locally advanced colon cancer remain limited. Comparable long-term outcomes observed in patients with T4 tumors provide compelling evidence for the feasibility of robotic surgery in complex resections when performed by experienced surgeons and substantiate its potential to expand minimally invasive approaches to more complex disease stages.

## Figures and Tables

**Table 1:** Comparison of demographic and therapy characteristics of patients undergoing ORH and RRH.

	Valid Cases	Total Cohort n = 198 Median [IQR] or Number (%) *	ORH n = 77 Median [IQR] or Number (%) *	RRH n = 121 Median [IQR] or Number (%) *	*p*-Value ^A^	d_Cohen_	Phi	Test Statistic	df/Z
Age [years]	198	75 [13]	75 [16]	76 [13]	0.767			4542	−0.297
Gender [female]	198	111 (56.1)							
BMI [kg m^−2^]	158	25 [6]	25 [5]	25 [7]	0.735			2386	−0.338
ASA	161	3 [1]	3 [1]	3 [1]	0.192			1.701	1
ECOG	156	1 [1]	1 [1]	1 [1]	0.249			1.327	1
Vascular disease ^a^	196	54 (27.3)	18 (24)	36 (30)	0.335			0.930	1
History of prior abdominal surgeries	195	109 (55.1)	42 (55.2)	67 (56.3)	0.887			0.020	1
OR time [min]	186	215 [93]	199 [99]	215 [93]	0.003	0.451		2994	−2.997
Blood loss [mL]	198	50 [0]	50 [0]	50 [0]	0.883			4620	−0.147
Blood transfusion intraoperative	198	7 (3.5)	2 (2.6)	5 (4.1)	0.569			0.325	1
Conversion	190	7 (3.6)	n/a	7 (6)	n/a				
Additional procedure	191								
Minor ^b^		26 (13.1)	16 (21.3)	10 (8.6)	0.013		0.181	6.227	1
Major ^c^		29 (14.6)	18 (24)	11 (9.5)	0.006		0.198	7.454	1
Intraoperative complication ^d^	198								
None		171 (86.4)	67 (87)	104 (86)	0.832				
Minor		27 (13.6)	10 (13)	17 (14)	0.832			0.045	1
Major ^e^		2 (0.5)	1 (1.3)	1 (0.8)	0.739			0.111	1
Establishing continuity	190	184 (92.9)	71 (95.9)	113 (97.4)	0.573			0.318	1
Stoma formation	198	14 (7.1)							
Peridural catheter	197	113 (57.1)	42 (54.5)	71 (59.2)	0.522			0.410	1
Chemotherapy	198	60 (30)	28 (36.4)	32 (26.4)	0.139				

* As appropriate. ^a^ Including peripheral artery disease, coronary artery disease. ^b^ Biopsy, atypical liver resection, intestinal suture. ^c^ Total or partial resection of other organs. ^d^ According to ClassIntra-Classification. ^e^ ClassIntra grade III or higher. ^A^ Statistics were realized by Fisher’s exact test, chi^2^ test, ANOVA, Mann–Whitney *U* test, or Kruskal–Wallis test, as appropriate. n/a: not applicable.

**Table 2:** Comparison of histopathological characteristics and survival between ORH and RRH.

	Valid Cases	Total Cohort n = 198 Median [IQR] or Number (%) *	ORH n = 77 Median [IQR] or Number (%) *	RRH n = 121 Median [IQR] or Number (%) *	*p*-Value ^A^	d_Cohen_	Phi	Test Statistic	df/Z
Entity	197				0.039			4.270	1
Carcinoma		189 (95.4)	76 (98.7)	113 (93.4)	0.021	0.3		5.305	1
Adenoma		8 (4.0)	0 (0)	8 (4.9)	0.021	0.3		5.305	1
UICC	191	2 [1]	2 [1]	2 [2]	0.109			2.570	1
UICC 0		7 (3.5)	3 (3.9)	4 (3.5)					
UICC I		39 (19.7)	11 (14.5)	28 (24.3)					
UICC II		74 (37.4)	29 (38.2)	45 (39.1)					
UICC III		55 (27.8)	26 (34.2)	29 (25.2)					
UICC IV		15 (7.6)	6 (7.9)	9 (7.8)					
T4		54 (27.3)	28 (36.4)	26 (21.8)	0.026		0.159	4.934	1
Lymph nodes	195								
Number resected		18 [10]	18 [9]	18 [9]	0.828			4394	−0.217
Number affected		0 [2]	0 [4]	0 [1]	0.088			3925	−1.706
R-Status	195				0.547			0.362	1
R0		189 (95.5)	72 (96)	117 (97.5)					
R1		5 (2.5)	2 (2.7)	3 (2.5)					
R2		1 (0.5)	1 (1.3)	0 (0)					
Tumor size (mm)	181	47 [29]	55 [28.5]	45 [29]	0.102			3218.5	−1.633
DFS ^a^	181	60 [32]	60 [47]	60 [18]	0.029	0.328		3258.5	−2.179
OS ^a^	189	60 [12]	60 [25]	60 [0]	0.13			3763	−1.584
Recurrence	176				0.271			1.211	1
Local		10 (5.1)	4 (5.7)	6 (5.7)					
Distant		12 (6.1)	12 (6.8)	5 (4.7)					

* As appropriate. ^a^ Data presented in months. ^A^ Statistics were realized by Fisher’s exact test, chi^2^ test, Mann–Whitney *U* test, or Kruskal–Wallis test, as appropriate.

**Table 3:** Comparison of postoperative outcome and complications between ORH and RRH.

	Valid Cases	Total Cohort n = 198 Median [IQR] or Number (%) *	ORH n = 77 Median [IQR] or Number (%) *	RRH n = 121 Median [IQR] or Number (%) *	*p*-Value ^A^	d_Cohen_	Phi	Test Statistic	df/Z
Length of stay									
ICU [d]	175	0 [1]	0 [2]	0 [0]	0.085			3221.5	−1.722
In total [d]	196	15 [11]	17 [9]	13 [8]	<0.001	0.626		2961.5	−4.182
Any complication ^a^	197								
Minor		58 (29.3)	21 (27.3)	37 (30.8)	0.593			0.286	1
Major		31 (15.7)	17 (22.1)	14 (11.7)	0.050		0.14	3.834	1
Clavien–Dindo complication score	197				0.149			2.080	1
I		9 (4.5)	3 (3.9)	6 (5)					
II		49 (24.7)	18 (23.4)	31 (25.8)					
IIIa		1 (0.5)	1 (1.3)	0 (0)					
IIIb		20 (10.1)	12 (15.6)	8 (6.7)					
IVa		5 (2.5)	1 (1.3)	4 (3.3)					
IVb		0 (0)	0 (0)	0 (0)					
V		5 (2.5)	3 (3.9)	2 (1.7)					
Blood transfusion in total	198	19 (9.6)	4 (5.2)	15 (12.4)	0.093			2.813	1
30-day mortality	197	6 (3.0)	4 (5.2)	2 (1.7)	0.160			1.977	1

* As appropriate. ^a^ According to Clavien–Dindo complication score. ^A^ Statistics were realized by Fisher’s exact test, chi^2^ test, Mann–Whitney *U* test, or Kruskal–Wallis test, as appropriate.

**Table 4:** Comparison of perioperative and postoperative outcomes between ORH and RRH in the T4 subgroup.

	Valid Cases	Total Cohort n = 54 Median [IQR] or Number (%) *	ORH n = 28 Median [IQR] or Number (%) *	RRH n = 26 Median [IQR] or Number (%) *	*p*-Value ^A^	d_Cohen_	Test Statistic	df/Z
Age [years]	54	74 [16]	74 [17]	74 [16]	0.709		342.5	−0.373
Gender [female]	54	32 (59.3)	20 (71.4)	12 (46.2)	0.059		3.567	1
BMI [kg m^−2^]	48	24 [6.8]	25 [5.4]	23 [7.9]	0.222		345	1.222
ASA	48	3 [1]	3 [1]	3 [1]	0.932		289.5	0.086
ECOG	42	2 [4]	2 [2]	2 [2]	0.979		211.5	−0.027
Chemotherapy	54	30 (55.6)	16 (57.1)	14 (53.8)	0.808		0.059	1
OR time [min]	52	208 [102]	195 [78]	215 [160]	0.468		296.5	−0.725
Blood loss [mL]	54	50 [0]	50 [0]	50 [0]	0.966		365.5	0.042
Conversion	53	4 (7.4)	n/a	4 (7.4)	n/a			
Additional procedure	53							
Minor ^a^		13 (24.1)	9 (32.1)	4 (16)	0.173		1.859	1
Major ^b^		16 (29.6)	10 (35.7)	6 (24)	0.354		0.860	1
Major Intraoperative complication ^c^	54	1 (1.9)	1 (3.6)	0 (0)	0.331		0.946	1
Length of stay								
ICU [d]	50	0 [2]	0 [2]	0 [3]	0.974		314	.033
In total [d]	54	18 [16]	20 [12]	14 [22]	0.012	0.727	509	2.514
Postoperative complication ^d^	54							
Minor		12 (22.2)	6 (21.4)	6 (23.1)	0.884		0.021	1
Major		14 (25.9)	9 (32.1)	5 (19.2)	0.279		1.170	1
30-Day mortality	54	3 (5.6)	2 (7.1)	1 (3.8)	0.597		0.279	1
Lymph nodes	53							
Number resected		19 [10]	18 [9]	21 [13]	0.266		288.5	−1.113
Number affected		2 [7]	1 [5]	2 [7]	0.771		335	−0.291
Tumor size (mm)	49	60 [29.5]	60 [30.5]	58 [30]	0.778		313	0.282
R0	54	49 (90.7)	25 (89.3)	24 (92.3)	0.679			
DFS ^e^	49	36 [48.5]	26 [48]	40.5 [52.2]	0.797		312.5	0.258
OS ^e^	50	60 [35.2]	60 [35]	60 [38.5]	0.77		326	0.292

* As appropriate. ^a^ Biopsy, atypical liver resection, intestinal suture. ^b^ Total or partial resection of other organs. ^c^ According to ClassIntra classification. ^d^ According to Clavien–Dindo complication score. ^e^ Data presented in months. ^A^ Statistics were realized by Fisher’s exact test, chi^2^ test, Mann–Whitney *U* test, or Kruskal–Wallis test, as appropriate, n/a: not applicable.

## Data Availability

The data that support the findings of this study are available from the corresponding author upon reasonable request.

## Supplementary Materials

The following supporting information can be downloaded at https://www.mdpi.com/article/10.3390/cancers18081310/s1; Table S1. Comparison of the details of demographic characteristics of patients undergoing ORH and RRH; Table S2. Comparison of preoperative and postoperative laboratory values between ORH and RRH; Table S3. Comparison of surgical procedure related complications between ORH and RRH; Table S4. Multivariable Cox proportional hazard regression analysis for overall survival (OS) in the entire cohort; Table S5. Multivariable Cox proportional hazards regression analysis for overall survival (OS) in the entire cohort, stratified by surgical approach (OP technique); Table S6. Multivariable Cox proportional hazards regression analysis for disease-free survival (DFS) in the entire cohort; Table S7. Multivariable Cox proportional hazard regression analysis for disease-free survival (DFS) in the entire cohort, stratified by surgical approach (OP technique); Table S8. Results of the binary logistic regression analysis for OS; Table S9. Results of the binary logistic regression analysis for DFS; Table S10. Log-rank Mantel–Cox analysis for OS and DFS; Figure S1. Kaplan–Meier curve for disease-free survival (DFS) in the T4 subgroup.

## Abbreviations

The following abbreviations are used in this manuscript:

CRC	Colorectal cancer
LRH	Laparoscopic right hemicolectomy
ORH	Open right hemicolectomy
RRH	Robotic right hemicolectomy
OS	Overall survival
DFS	Disease-free survival
MIS	Minimally invasive surgery
CME	Complete mesocolic excision
BMI	Body mass index
ASA	American Society of Anesthesiologists
ECOG	Eastern Cooperative Oncology Group
UICC	Union for International Cancer Control
BL	Blood loss
PDC	Peridural catheter
LOS	Length of (hospital) stay
