# Supplementary material for: Robotic Right Hemicolectomy Provides Equivalent Oncologic Outcomes and Improved Perioperative Recovery Compared with Open Surgery
Source: Cancers (Basel). 2026 Apr 21;18(8):1310. doi: 10.3390/cancers18081310 (PMC13114466; doi:10.3390/cancers18081310)
Supplement: Supplementary file 1 [file cancers-18-01310-s001.zip › cancers-4228623-supplementary.pdf]

Title: Robotic right hemicolectomy provides equivalent oncologic outcomes and partly improved perioperative recovery compared with open surgery even in locally advanced colon cancer

Hatice Altin[1], Thorsten Brechmann[1 2], Methin Mazgaldzhi[1 4], Anna-Marie Wilk[3], Benno Mann[1 4], Alexander Wilk[1 4]

Affilitations:

1 Ruhr-Universität Bochum (Bochum, Germany)

2 Knappschaft Kliniken Bottrop, Department of Gastroenterology and Oncology (Bottrop, Germany)

3 Wroclaw Medical University (Wroclaw, Poland)

4 Augusta-Klinikum Bochum Mitte, Clinic for General, Visceral and Robotic Surgery (Bochum, Germany)

Address for correspondence:

Alexander Wilk, Augusta-Kranken-Anstalt gGmbH, Bochum-Mitte, Bergstraße 26, 44791 Bochum. Email: a.wilk@augusta-kliniken.de. Tel.: +49 (0) 234 517-0, Fax: +49 (0) 234 517-1372

Supplementary Materials - Index

Supplementary Tables

Table S1. Comparison of the details of demographic characteristics of patients undergoing ORH and RRH — ***pag. 3***

Table S2. Comparison of preoperative and postoperative laboratory values between ORH and RRH — ***pag.4***

Table S3. Comparison of surgical procedure related complications between ORH and RRH — ***pag. 5***

Table S4. Multivariable Cox proportional hazards regression analysis for overall survival (OS) in the entire cohort — ***pag. 6***

Table S5. Multivariable Cox proportional hazards regression analysis for overall survival (OS) in the entire cohort, stratified by surgical approach (OP-technique) *pag. 7*

Table S6. Multivariable Cox proportional hazards regression analysis for disease free survival (DFS) in the entire cohort *pag. 8*

Table S7. Multivariable Cox proportional hazards regression analysis for disease free survival (DFS) in the entire cohort, stratified by surgical approach (OP-technique) *pag. 9*

Table S8. Results of the binary logistic regression analysis for OS *pag. 10*

Table S9. Results of the binary logistic regression analysis for DFS *pag. 11*

Table S10. Log Rank Mantel-Cox Analysis for OS and DFS *pag. 12*

Figure S1. Kaplan-Meier curve for disease free survival (DFS) in the T4 subgroup *pag. 13*

Supplementary Tables

Table S1. Comparison of the details of demographic characteristics of patients undergoing ORH and RRH

	**Valid cases**	**Total Cohort n = 198** Median [IQR] or number (%)*	**ORH n = 77** Median [IQR] or number (%)*	**RRH n = 121** Median [IQR] or number (%)*	***p*-value**[A]	**Test statistic**	**df/Z**
**ASA**	161	3 [1]	3 [1]	3 [1]	.192	1.701	1
1		2 (1.0)	1 (2.1)	1 (0.9)			
2		54 (27.3)	12 (25)	42 (37.2)			
3		100 (50.5)	33 (68.8)	67 (59.3)			
4		5 (2.5)	2 (4.2)	3 (2.7)			
**ECOG**	156	1 [1]	1 [1]	1 [1]	.249	1.327	1
0		34 (17.2)	3 (6.8)	31 (27.7)			
1		50 (25.3)	19 (43.2)	31 (27.7)			
2		44 (22.2)	16 (36.4)	28 (25)			
3		26 (13.1)	6 (13.6)	20 (17.9)			
4		2 (1.0)	0 (0)	2 (1.8)			

* As appropriate
[A] Statistics were realised by Fisher's exact test, ANOVA or Mann-Whitney-*U*-Test, as appropriate

Table S2. Comparison of preoperative and postoperative laboratory values between ORH and RRH

	Valid cases	Total Cohort n = 198 Median [IQR] or number (%)*	ORH n = 77 Median [IQR] or number (%)*	RRH n = 121 Median [IQR] or number (%)*	*p*-value[A]	Test statistic	df/Z
**Pre-Operative**							
Leukocytes (cells/ul)	153	7.5 [3.2]	7.3 [3]	7.6 [3.3]	.807	2831.5	-.244
Hemoglobin [g/dL]	153	10 [3]	10.1 [2.7]	9.8 [3.6]	.561	2739.5	-.581
Thrombocytes [/ul]	153	293 [142]	302 [153]	280 [136]	.445	2689.5	-.765
Quick	153	93 [17]	93 [18]	93 [17]	.934	2867.5	-.083
Creatinine [mg/dl]	153	0.9 [0.5]	0.9 [0.5]	0.9 [0.4]	.314	2617.5	-1.007
CRP (mg/L)	152	0.8 [2.5]	1.35 [3.2]	0.55 [1.2]	.051	2331.5	-1.949
CEA (ng/mL)	80	3.0 [9]	3.7 [14.1]	2.8 [4.7]	.333	661.5	-.968
CA-19.9 (ng/mL)	80	12.8 [16.4]	18.5 [35.5]	11.5 [16.3]	.059	568.5	-1.887
**Post-Operative**							
CEA [ng/mL]	8	2.7 [14.2]	2.7 [177]	7.6 [n/a]	.867	5.500	-.168
CA-19.9 (ng/mL)	7	10.6 [20]	17.8 [2627]	6.3 [n/a]	.699	4.000	-.387

* As appropriate
[A] Statistics were realised by Fisher's exact test, ANOVA or Mann-Whitney-*U*-Test, as appropriate

Table S3. Comparison of surgical procedure related complications between ORH and RRH

	Valid cases	Total Cohort n = 198 Median [IQR] or number (%)*	ORH n = 77 Median [IQR] or number (%)*	RRH n = 121 Median [IQR] or number (%)*	*p*-value[A]	Test statistic	df/Z
**Surgical Complications[a]**	197	49 (24.7)	22 (28.6)	27 (22.5)	.336	.925	1
**Anastomotic leak[b]**	196				.276	3.866	3
Grad A		2 (1.0)	2 (1)	0 (0)			
Grad B		1 (0.5)	0 (0)	1 (0.5)			
Grad C		12 (6.1)	5 (2.6)	7 (3.6)			
**Non-surgical Complications[c]**	197	62 (31.3)	28 (36.4)	34 (28.3)	.236	1.402	1
**Re-Operation**	196	25 (12.6)	14 (12.8)	11 (9.2)	.058	3.581	1

[a] Including bleeding, atony, anastomosis insufficiency, incisional hernia, wound infection, ileus, injury to other organs, ischemia, blood transfusion
[b] according to ISREC Classification
[c] Including DVT, PE, UTI, pneumonia, pleural effusion, electrolyte disturbances
[A] Statistics were realised by Fisher's exact test, Chi^2 test, Man-Whitney U-Test or Kruskal-Wallis-test, as appropriate

Table S4. Multivariable Cox proportional hazards regression analysis for overall survival (OS) in the entire cohort

Model: Entire cohort
Outcome: Overall survival
Model type: Adjusted Cox proportional hazards model

Variables in the equation	β (Coefficient)	SE	Wald χ^2	df	HR (Exp(B))	95% CI (HR)	*p*-value
**Chemotherapy (neoadjuvant)**	-.346	.631	.300	1	.708	.206 - 2.437	.584
**Minor additional procedure**	-.474	.408	1.347	1	.623	.280 - 1.386	.246
**Major additional procedure**	-.073	.451	.026	1	.929	.384 - 2.248	.871
**Chemotherapy (adjuvant)**	.174	.341	.261	1	1.191	.610 - 2.324	.609
**T4**	-.579	.348	2.766	1	.560	.283 - 1.109	.096
**OP-Technique**	-.209	.316	.438	1	.811	.436 - 1.508	.508
**R-Status**	-1.655	.592	7.817	1	.191	.060 - .610	**.005**

Results are presented as β-coefficients, standard errors (SE), hazard ratios (HR), 95% confidence intervals (CI), and p-values

Table S5. Multivariable Cox proportional hazards regression analysis for overall survival (OS) in the entire cohort, stratified by surgical approach (OP-technique)

Model: Entire cohort
Stratification: OP-Technique
Outcome: Overall survival
Model type: Adjusted Cox proportional hazards model

Variables in the equation	β (Coefficient)	SE	Wald χ^2	df	HR (Exp(B))	95% CI (HR)	*p*-value
**ECOG**	.299	.182	2.684	1	1.348	.943 - 1.928	.101
**Chemotherapy(total)**	-.020	.391	.003	1	.980	.455 - 2.110	.959
**Minor additional procedure**	.075	.484	.024	1	1.078	.418 - 2.780	.877
**Major additional procedure**	.279	.497	.316	1	1.322	.499 - 3.504	.574
**R-Status**	1.569	.777	4.080	1	4.803	1.048 – 22.020	**.043**
**T4**	.365	.404	.817	1	1.440	.653 – 3.177	.366
**Lymphnodes (affected)**	.027	.028	.933	1	1.028	.972 – 1.086	.334
**Major postoperative complication**	.441	.425	1.076	1	1.554	.676 – 3.574	.299

Results are presented as β-coefficients, standard errors (SE), hazard ratios (HR), 95% confidence intervals (CI), and p-values

Table S6. Multivariable Cox proportional hazards regression analysis for disease free survival (DFS) in the entire cohort

Model: Entire cohort
Outcome: Disease free survival (recurrence)
Model type: Adjusted Cox proportional hazards model

Variables in the equation	β (Coefficient)	SE	Wald χ^2	df	HR (Exp(B))	95% CI (HR)	*p*-value
**Chemotherapy (neoadjuvant)**	.293	1.028	.081	1	1.340	.179 - 10.049	.776
**Minor additional procedure**	-.739	.373	3.925	1	.478	.230 - .992	.048
**Major additional procedure**	-1.114	.349	10.195	1	.328	.166 - .650	**.001**
**Chemotherapy (adjuvant)**	-.098	.302	.105	1	.907	.502 - 1.639	.746
**T4**	-.968	.302	10.251	1	.380	.210 - .687	**.001**
**OP-Technique**	.067	.289	.054	1	1.069	.607 - 1.883	.816
**R-Status**	-.473	.590	.641	1	.423	.196 - 1.983	.423

Results are presented as β-coefficients, standard errors (SE), hazard ratios (HR), 95% confidence intervals (CI), and p-values

Table S7. Multivariable Cox proportional hazards regression analysis for disease free survival (DFS) in the entire cohort, stratified by surgical approach (OP-technique)

Model: Entire cohort
Stratification: OP-Technique
Outcome: Disease free survival (recurrence)
Model type: Adjusted Cox proportional hazards model

Variables in the equation	β (Coefficient)	SE	Wald χ^2	df	HR (Exp(B))	95% CI (HR)	*p*-value
**ECOG**	-.007	.174	.002	1	.993	.706-1.397	.967
**Chemotherapy(total)**	-.035	.376	.009	1	.965	.462 - 2.017	.925
**Minor additional procedure**	.428	.459	.870	1	1.534	.624 - 3.771	.351
**Major additional procedure**	1.176	.403	8.526	1	3.242	1.472 – 7.139	**.004**
**R-Status**	-.095	.763	.015	1	.910	.204 – 4.056	.901
**T4**	1.125	.379	8.786	1	3.080	1.464 – 6.478	**.003**
**Lymphnodes (affected)**	.047	.027	3.001	1	1.048	.994 – 1.105	.083
**Major postoperative complication**	.621	.425	2.137	1	1.860	.809 – 1.105	.144
Results are presented as β-coefficients, standard errors (SE), hazard ratios (HR), 95% confidence intervals (CI), and p-values							

Table S8. Results of the binary logistic regression analysis for OS

	B	SE	Wald	*p*-value[A]	OR	CI for OR lower bound	CI for OR upper bound
**BMI** [kg m^{-2}]	.024	.037	.441	.507	1.025	.953	1.101
**ASA**	-22.176	40193.265	≤ .001	1	≤ .001	≤ .001	n/a
**ECOG**	1.004	.761	1.741	.187	.526	.094	2.957
**Additional procedures**							
Minor	.407	.727	.313	.576	1.502	.361	6.251
Major	.819	.786	1.084	.298	2.267	.486	10.586
**Major postoperative complication**[a]	.821	.639	1.651	.199	2.273	.649	7.956
**Chemotherapy**	.004	.645	≤ .001	.995	.996	.281	3.53
**T4**	.594	.673	.779	.377	1.811	.485	6.769
**Lymphnodes affected**	.034	.077	.196	.658	1.035	.89	1.203
**R-Status**	22.309	19908.289	≤ .001	.999	4880463397	≤ .001	n/a
**Tumorsize**	.008	.01	.787	.375	.992	.973	1.01

[a] according to Clavien-Dindo-Complication-Score
[A] Statistics were realised by binary logistic regression analysis
OR: Odds ratio
CI: Confidence intervall
n/a: not applicable

Table S9. Results of the binary logistic regression analysis for DFS

	B	SE	Wald	*p*-value[A]	OR	CI for OR lower bound	CI for OR upper bound
**BMI** [kg m^{-2}]	.062	.039	2.568	.109	1.064	.986	1.149
**ASA**	-23.219	40192.764	≤ .001	1	≤ .001	≤ .001	n/a
**ECOG**	.231	.856	.073	.787	1.26	.235	6.748
**Additional procedures**							
Minor	.7	.711	.97	.325	2.014	.5	8.115
Major	2.541	.902	7.937	**.005**	12.688	2.167	74.3
**Major postoperative complication[a]**	.806	.695	1.347	.246	2.239	.574	8.735
**Chemotherapy**	-.209	.674	.096	.757	.811	.216	3.043
**T4**	1.95	.681	8.202	**.004**	7.031	1.851	26.714
**Lymphnodes affected**	.019	.064	.089	.765	1.019	.899	1.156
**R-Status**	.671	1.677	.16	.689	1.155	.191	3.033
**Tumorsize**	.004	.009	.192	.661	1.004	.986	1.022

[a] according to Clavien-Dindo-Complication-Score
[A] Statistics were realised by binary logistic regression analysis
OR: Odds ratio
CI: Confidence intervall
n/a: not applicable

Table S10. Log Rank Mantel-Cox Analysis for OS and DFS

	Chi-Square	df	p-value
**DFS**	.083	1	.773
**OS**	.031	1	.861

Survival distributions were compared using the log-rank (Mantel-Cox) test
Data are presented as Chi-Square test statistics with corresponding degrees of freedom (df) and p-values

Figure S1. Kaplan-Meier curve for disease free survival (DFS) in the T4 subgroup

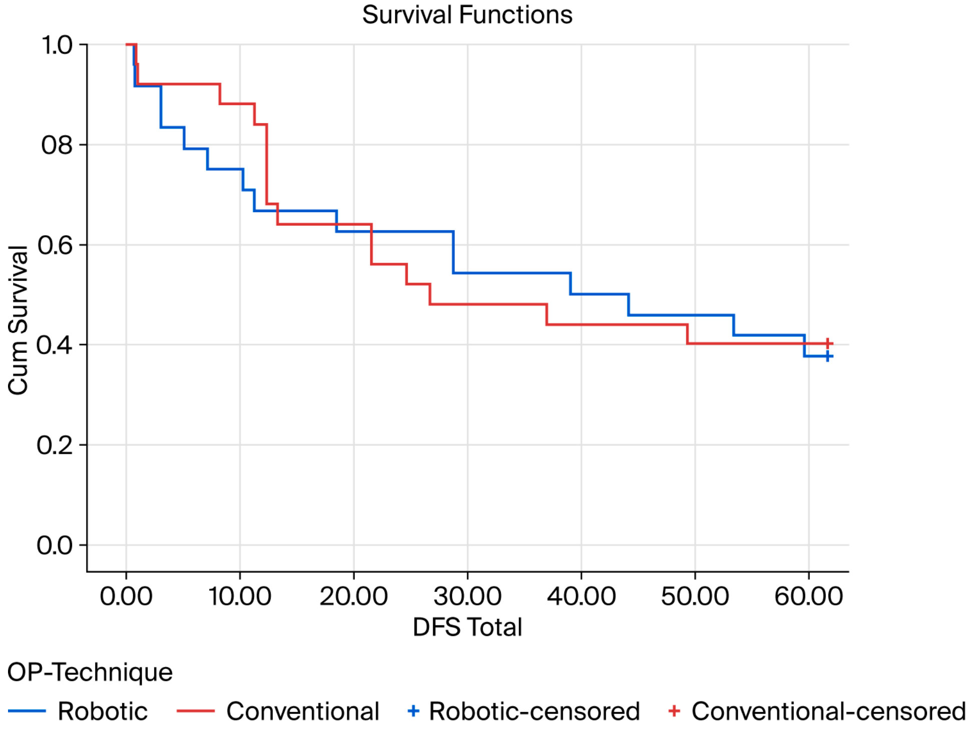

Time (months)	0	12	24	36	48	60
ORH	24	17	13	11	10	9
RRH	23	16	15	13	11	8

Kaplan-Meier survival analysis for DFS – T4 subgroup ORH: 26 months [IQR 48.0] vs. RRH: 40.5 months [52.2]; p = 0.797; Chi-Square = 0.031; p = 0.86

DFS = Disease free survival, ORH = Open right hemicolectomy, RRH = Robotic right hemicolectomy
